# Cognitive Mechanisms Reciprocally Transmit Vulnerability between Depressive and Somatic Symptoms

**DOI:** 10.1155/2015/250594

**Published:** 2015-12-09

**Authors:** Kaitlin A. Harding, Karly M. Murphy, Amy Mezulis

**Affiliations:** Seattle Pacific University, Marston 107, 3307 3rd Avenue West, Seattle, WA 98119-1922, USA

## Abstract

Despite high comorbidity between depressive and somatic symptoms, cognitive mechanisms that transmit vulnerability between symptom clusters are largely unknown. Dampening, positive rumination, and brooding are three cognitive predictors of depression, with rumination theoretically indicated as a transdiagnostic vulnerability through amplifying and diminishing affect in response to events. Specifically, the excess negative affect and lack of positive affect characteristic of depressive symptoms and underlying somatic symptoms may cause and be caused by cognitive responses to events. Therefore, the current study examined whether comorbidity between depressive and somatic symptoms may be explained by the cognitive mechanisms of dampening and positive rumination in response to positive events and brooding in response to negative events among adults (*N* = 321) across eight weeks of assessment. We hypothesized that greater dampening and brooding would reciprocally predict greater depressive and somatic symptoms, while greater positive rumination would reciprocally predict fewer depressive and somatic symptoms. Mediation analyses in AMOS 22 indicated that dampening and brooding mediated reciprocal pathways between depressive and somatic symptoms, but positive rumination did not. Findings propose dampening and brooding as mechanisms of the reciprocal relationship between depressive and somatic symptoms through diminishing positive affect and amplifying negative affect in response to positive and negative events.

## 1. Introduction

Affective and somatic symptoms are common and debilitating categories of psychological health concerns in the United States, representing key causes of distress and impaired functioning across the lifespan. Somatic symptoms frequently cooccur with affective difficulties including anxiety, trauma-related, and depressive symptoms [1–3]. Of these comorbidities, cooccurring depressive and somatic symptoms may be of particular clinical and research concern. Research organizations including the World Health Organization describe both depressive and somatic symptoms as socially burdensome mental disorders globally and important focuses for research across nationalities and demographics [4–6]. An international investigation of comorbid depressive and somatic symptoms across 15 countries determined that 30–62% of individuals with a Major Depressive Disorder diagnosis also reported somatic symptoms that were not explained by a known physical etiology [7], which may be partially explained by the somatic nature of certain depressive symptoms. Depressive and somatic symptoms are highly comorbid, with neither symptom category consistently preceding the onset of the other [8].

Despite the established relationship between somatic and depressive symptoms, little is known regarding mechanisms that may explain the transmission of vulnerability between somatic and depressive symptoms. However, cognitive theories for both symptom clusters suggest that how individuals think in response to events may explain shared pathways and shared health outcomes for these commonly cooccurring symptoms. Applied practically, understanding shared cognitive vulnerabilities between symptom clusters may highlight ways that individuals internally process social events and thereby inform clinical interventions to decrease symptoms and increase engagement. For example, identifying transdiagnostic cognitive mechanisms may encourage clinicians to generalize depressive symptom interventions such as mindfulness-based cognitive therapy [9] to physical concerns such as fibromyalgia, gastrointestinal distress, and chronic fatigue syndrome [10]. Identifying transdiagnostic vulnerabilities also may further justify and inform interventions that treat multiple concerns simultaneously, such as depressive and somatic symptom comorbidity. As health care increasingly integrates psychological and physical health conceptualization, the need to understand reciprocal transmission between symptom clusters is becoming an interdisciplinary agenda [11]. Depressive and somatic symptoms represent prevalent and comorbid concerns across health settings, and understanding the interplay of symptoms is a crucial step toward integrating health care as a holistic mind-body endeavor. As a step toward integration, the current study examined the role of three cognitive mechanisms that may reciprocally explain the comorbidity between depressive and somatic symptom clusters using a multiwave study design.

Cognitive theories suggest that affective responses elicited by positive and negative events are amplified or diminished by cognitive responses to those events, which over time contribute to depressive and somatic symptoms [12–15]. Parallel theoretical explanations of cognitive responses as pathways to depressive and somatic symptoms suggest shared vulnerabilities through cognitive responses to positive and negative events. However, cognitive responses to positive events are unexamined as predictors of somatic symptoms. Similarly, reciprocal cognitive pathways between depressive and somatic symptoms are unknown. Because depressive and somatic symptoms frequently predict each other, shared cognitive pathways may reciprocally explain the comorbidity between depressive and somatic symptom clusters as well as proposing cognitive targets for prevention and treatment.

Cognitive theories on depressive and somatic symptoms propose cognitive mechanisms in the reciprocal transmission of vulnerability between symptom clusters. Based on previous literature, the cognitive responses of dampening, positive rumination, and brooding are three cognitive vulnerabilities to depression. Dampening describes thoughts that decrease positive affect in response to positive events [16], which predicts greater depressive symptoms above and beyond rumination in response to negative events [17]. Examples of dampening include thinking about the potential negative consequences of a friendly social interaction or distracting attention away from an enjoyable conversation. As a counterpart to dampening, positive rumination describes thoughts that increase positive affect in response to positive events [18], which predicts fewer depressive symptoms and negatively correlates with rumination in response to negative events [19]. Examples of positive rumination include focusing attention on personal strengths or thinking about a pleasurable experience you shared with a friend. Finally, brooding describes thoughts that increase negative affect in response to negative events [20], which predicts greater depressive symptoms distinctly from dampening and positive rumination [19]. Examples of brooding include repeatedly thinking about whether a person dislikes you based on a past interaction or whether you embarrassed yourself during a public speaking event. Brooding is a type of rumination on negative content while positive rumination is a type of rumination on positive content.

Although the specific cognitive responses of dampening, positive rumination, and brooding have not been examined as predictors of somatic symptoms, rumination in general is indicated as a cognitive vulnerability to somatic symptoms [21–23]. Rumination describes perseverative focus on positive or negative affective content, which over time amplifies the affective content of focus [24]. As two distinct yet related symptom clusters, rumination on positive events through positive rumination and rumination on negative events through brooding may represent joint cognitive mechanisms that explain depressive and somatic symptom comorbidity by adaptively increasing positive affect in response to positive events or maladaptively increasing negative affect in response to negative events. As a potential explanation for this vulnerability, Brosschot and colleagues [14] proposed that perseverative thoughts prolong stress-related affect and physiological arousal due to stressful events, which over time predict somatic symptoms. Consistent with this model, a number of studies indicate that ruminative thinking in response to negative events is related to perceived physical symptoms [25–27]. Taken together, individuals who dampen and brood in response to events may be more likely to develop depressive and somatic symptoms, while individuals who positively ruminate may be less likely to develop symptoms.

**Figure 1:**
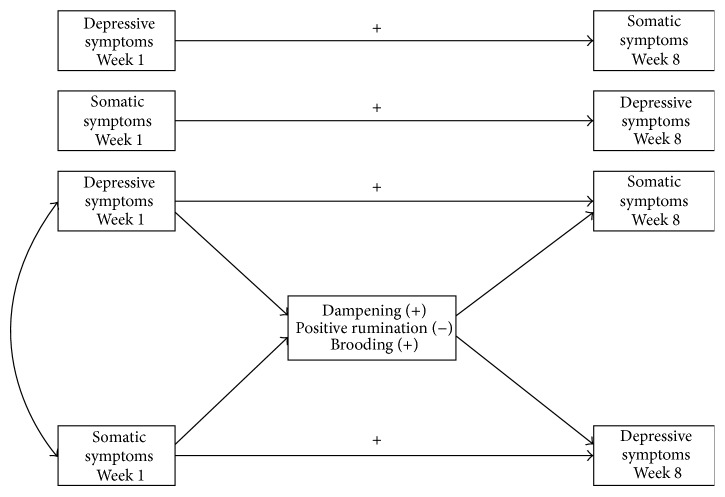
Theoretical model of the reciprocal relationships between depressive and somatic symptoms as mediated by dampening, positive rumination, and brooding.

The current study examined bidirectional relationships between depressive and somatic symptoms as mediated by dampening and positive rumination in response to positive events and brooding in response to negative events (see Figure 1) in a short-term prospective study among young adults. Recent studies comparing adults with and without chronic medical conditions demonstrated that younger adults reported greater psychological distress compared to older adults [28, 29]. As a result, young adults may be more vulnerable to psychological distress such as depressive symptoms when comorbid somatic symptoms are also present. To further examine the relationships between depressive and somatic symptoms among young adults specifically, we hypothesized that depressive and somatic symptoms would reciprocally predict each other across the eight-week study period. As an extension of existing literature, we further hypothesized that dampening, positive rumination, and brooding would mediate the reciprocal relationships between depressive and somatic symptoms. First, we hypothesized that greater dampening would reciprocally predict greater depressive and somatic symptoms. Second, we hypothesized that greater positive rumination would reciprocally predict fewer depressive and somatic symptoms. Third, we hypothesized that greater brooding would reciprocally predict greater depressive and somatic symptoms.

## 2. Materials and Methods

### 2.1. Participants and Procedure

Participants were 321 (73.52% female) undergraduate students recruited from a university in the Pacific Northwest who were 18–29 years old with a mean age of 19.03 years (SD = 1.64 years). Approximately 70.40% of participants were Caucasian American, 2.80% were African American, 16.51% were Asian American, 0.62% were Native American, 4.67% were Hispanic/Latin American, and 5.00% were identified as another or multiple cultural backgrounds.

Participants were recruited in undergraduate psychology courses across two academic years to complete a baseline questionnaire that included measures of demographic information, depressive symptoms, and somatic symptoms. Participants who completed the baseline questionnaire were then invited to participate in the next six weekly questionnaires of the study, which included measures of cognitive responses to positive and negative events. Finally, participants completed a final questionnaire measuring depressive and somatic symptoms at week eight. Participants received course research credit and were able to complete each questionnaire during a 48-hour period each week to maintain an interval of approximately one week between all eight questionnaires.

### 2.2. Measures

#### 2.2.1. Depressive Symptoms

Depressive symptoms over the past week were measured at weeks one and eight with the Center for Epidemiologic Studies Depression Scale (CES-D, [30]), which is a 20-item measure of depressive symptoms over the past week. The CES-D was selected because it uniquely measures emotional and cognitive depressive symptoms without overlapping with physical symptoms that are shared by depressive and somatic symptoms. The CES-D also includes all criteria for Major Depressive Disorder according to the most recent* Diagnostic and Statistical Manual of Mental Disorders, Fifth Edition* (DSM-V, [8]), except for suicidality and recurrent thoughts of death. Participants rated to what extent they experienced each item from 0 (*rarely or none of the time*) to 3 (*most or all of the time*) for items such as the following: “I was bothered by things that usually don't bother me” and “I felt lonely.” A mean score was calculated, with higher scores representing greater depressive symptoms over the past week. Cronbach's alpha coefficient for the CES-D was 0.85 in a nonclinical sample similar to our own and demonstrated strong convergent validity with other measures of depressive symptoms [30]. In our study, Cronbach's alpha coefficients for the CES-D were 0.88 at week one and 0.90 at week eight.

#### 2.2.2. Somatic Symptoms

Somatic symptoms over the past week were measured at weeks one and eight with the Modified Somatic Perception Questionnaire (MSPQ, [31]), which is a 13-item measure of a person's experience of somatic symptoms over the past week. The MSPQ was selected because it measures physical symptoms associated with psychological distress without overlapping emotional or cognitive item content with the CES-D. The MSPQ also measures somatic perceptions rather than specific medical symptoms or conditions, which allows consideration of physical concerns that may not be explained by known medical conditions. All items represent prevalent and medically relevant physical symptoms that are not attributed to a specific cause. Participants rated to what extent they experienced each item from 1 (*not at all*) to 4 (*extremely/could not have been worse*) for items such as “nausea” and “feeling tense across the forehead.” A mean score was calculated, with higher scores representing greater somatic symptoms over the past week. Cronbach's alpha coefficient for the MSPQ ranged from 0.78 to 0.85 across previous studies [31]. In our study, Cronbach's alpha coefficients for somatic symptoms were 0.83 at week one and 0.88 at week eight.

#### 2.2.3. Event-Specific Dampening

Dampening in response to a specific positive event over the past week was measured across weeks two through seven with the three highest loading items of the dampening subscale of the Responses to Positive Affect Scale (RPA, [19]), which measures a participant's tendency to dampen in response to the most positive event over the past week. The RPA was selected because it was the only psychometrically validated measure of dampening available at the time of our study. Participants rated to what extent they did or thought about each item from 1 (*almost never*) to 4 (*almost always*) for items such as the following: “think about the things that have not gone well for you.” A mean score was calculated across weeks two through seven, with higher scores representing greater event-specific dampening over the past week. Cronbach's alpha coefficient for RPA dampening was 0.79 in a previous study [19]. In our study, Cronbach's alpha coefficients for dampening ranged from 0.70 to 0.80 across weeks.

#### 2.2.4. Event-Specific Positive Rumination

Positive rumination in response to a specific positive event over the past week was measured across weeks two through seven with the three highest loading items of the positive rumination subscales of the RPA [19], which measure a participant's tendency to positively ruminate in response to the most positive event over the past week. The RPA was selected because it was the only psychometrically validated measure of positive rumination available at the time of our study and it includes a dampening measure using the same scale. Participants rated to what extent they did or thought about each item from 1 (*almost never*) to 4 (*almost always*) for items such as the following: “think about how proud you are of yourself.” A mean score was calculated across weeks two through seven, with higher scores representing greater event-specific positive rumination over the past week. Cronbach's alpha coefficients for RPA positive rumination ranged from 0.73 to 0.76 across previous studies [19]. In our study, Cronbach's alpha coefficients for positive rumination ranged from 0.79 to 0.89 across weeks.

#### 2.2.5. Event-Specific Brooding

Brooding in response to a specific negative event over the past week was measured across weeks two through seven with the brooding subscale of the Ruminative Responses Scale (RRS, [24]), which is a 5-item measure of ruminative responses in response to the most negative event over the past week. The RRS was selected because it is a widely used and supported measure that facilitates a standardized comparison of brooding across studies that similarly utilize the RRS. The brooding subscale was proposed by Treynor et al. [20] and corroborated by Armey et al. [32] as a significant cognitive contributor to the generation of depressive symptoms. Participants rated how often they experienced each item from 1 (*never*) to 4 (*always*) for items such as the following: “think ‘what am I doing to deserve this?'” and “think about a recent situation, wishing it had gone better.” A mean score was calculated across weeks two through seven, with higher scores representing greater event-specific brooding over the past week. Cronbach's alpha coefficients for brooding ranged from 0.72 to 0.78 across nonclinical samples [20, 33]. In our study, Cronbach's alpha coefficients for brooding ranged from 0.81 to 0.86 across weeks.

### 2.3. Data Analytic Plan

Missing data analyses in SPSS 21 indicated 0.94% of our data were missing across weeks. Data were missing completely at random (MCAR) for all weeks except weeks one and five as indicated by nonsignificant Little's MCAR tests (*p* = 0.19 to 0.86). Week one missingness was determined as nonrandom because there was no missing data for any MSPQ items but there was 0.44% missing data for the CES-D. Week five was determined as nonrandom because eight participants ended the questionnaire early. Due to the minimal missingness accounting for the significant values, data were considered MCAR overall and multiply imputed to maximize our power to detect significant effects.

We conducted mediation analyses across eight weeks through AMOS 22, which allowed us to investigate causal relationships with two independent variables (i.e., depressive and somatic symptoms at week 1) predicting two dependent variables (i.e., depressive and somatic symptoms at week 8) through three independent variables (i.e., dampening, positive rumination, and brooding) within a single mediation model. Bootstrap resampling was conducted to test for the significance of indirect effects for all three cognitive mediators, which is proposed to maximize statistical power by computing nonsymmetric confidence intervals and reducing Type II error [34]. We generated 1,000 bootstrap samples with 95% bias-corrected confidence intervals and bootstrap estimates of indirect, direct, and total effects.

## 3. Results

**Figure 2:**
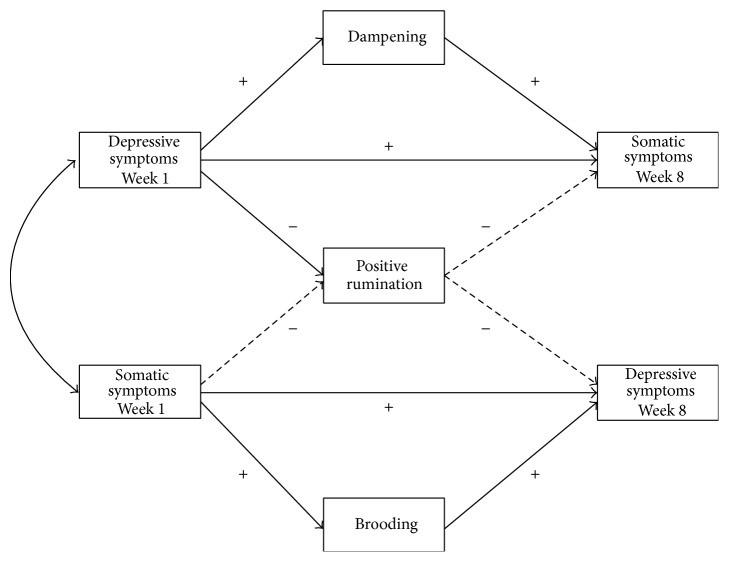
Relationships between depressive and somatic symptoms as mediated by dampening, positive rumination, and brooding. Dashed lines indicate nonsignificance.

Variablecorrelations and descriptors are presented in Table 1. Analyses controlled for the symptom cluster at baseline that was predicted seven weeks later to ensure that the effects of depressive and somatic symptoms were distinct. We ran analyses with all mediators simultaneously entered into a combined model to ensure that the unique effect of each cognitive response was not confounded by shared variance with the other two cognitive responses. Mediation results are presented in Tables 2 and 3, and significant pathways are graphically depicted in Figure 2. In both tables, the combined model section indicates the total effect (*c*) and the direct effect (*c*′) of the model that includes the combined effects of all three mediators. Combined *c*′ controls for the effect of all three proposed mediators simultaneously. The remaining sections separately provide *α*, *β*, *c*′, and indirect effects (*α* × *β*) of each mediator. To calculate *c*′ of each mediator, we ran analyses that distinctly controlled for the effect of each mediator in the relationship between depressive and somatic symptoms. Combined *c*′ reports the pooled variance contributed by the three mediators together, while *c*′ for each mediator reports the unique variance contributed by each mediator.

### 3.1. Do Depressive and Somatic Symptoms Reciprocally Predict Each Other?

We first examined whether greater depressive symptoms predicted greater somatic symptoms across the eight-week study period. Consistent with previous research, results indicated a significant relationship between baseline depressive and somatic symptoms seven weeks later, even after controlling for baseline somatic symptoms. We then examined whether greater somatic symptoms predicted greater depressive symptoms across the study period. Also consistent with previous research, results indicated a significant relationship between baseline somatic symptoms and depressive symptoms seven weeks later, even after controlling for baseline depressive symptoms.

### 3.2. Does Greater Dampening Reciprocally Predict Greater Depressive and Somatic Symptoms?

We examined whether greater somatic symptoms predicted greater depressive symptoms as mediated by greater dampening in response to weekly positive events. As hypothesized, results indicated that greater baseline somatic symptoms significantly predicted greater dampening, greater dampening significantly predicted greater depressive symptoms at week eight, and the effect of somatic symptoms on depressive symptoms significantly decreased when dampening was entered into the model. Specifically, the effect of baseline somatic symptoms on depressive symptoms at week eight decreased by 19.48% when dampening was in the model, suggesting that the effect of somatic symptoms on depressive symptoms is partially mediated by greater dampening in response to weekly positive events.

We then examined whether greater depressive symptoms predicted greater somatic symptoms as mediated by greater dampening in response to weekly positive events. Similarly, results indicated that greater baseline depressive symptoms significantly predicted greater dampening, greater dampening significantly predicted greater somatic symptoms at week eight, and the relationship between depressive and somatic symptoms significantly decreased when dampening was entered into the model. Specifically, the effect of baseline depressive symptoms on somatic symptoms at week eight decreased by 23.26% when dampening was in the model, suggesting that the effect of somatic symptoms on depressive symptoms is partially mediated by greater dampening in response to weekly positive events.

### 3.3. Does Greater Positive Rumination Reciprocally Predict Fewer Depressive and Somatic Symptoms?

We examined whether fewer somatic symptoms predicted fewer depressive symptoms as mediated by greater positive rumination in response to weekly positive events. Results indicated that fewer baseline somatic symptoms did not significantly predict greater positive rumination, and greater positive rumination did not significantly predict fewer depressive symptoms at week eight. We then examined whether fewer depressive symptoms predicted fewer somatic symptoms as mediated by greater positive rumination in response to weekly positive events. Results indicated that fewer baseline depressive symptoms significantly predicted greater positive rumination, but greater positive rumination did not significantly predict fewer somatic symptoms at week eight. Contrary to hypotheses, positive rumination did not mediate the relationship between depressive and somatic symptoms in either direction.

### 3.4. Does Greater Brooding Reciprocally Predict Greater Depressive and Somatic Symptoms?

Finally, we examined whether greater somatic symptoms predicted greater depressive symptoms as mediated by greater brooding in response to weekly negative events. As hypothesized, results indicated that greater baseline somatic symptoms significantly predicted greater brooding, greater brooding significantly predicted greater depressive symptoms at week eight, and the effect of somatic symptoms on depressive symptoms significantly decreased when brooding was entered into the model. Specifically, the effect of baseline somatic symptoms on depressive symptoms at week eight decreased by 19.48% when brooding was in the model, suggesting that the effect of somatic symptoms on depressive symptoms is partially mediated by greater brooding in response to weekly negative events.

We then examined whether greater depressive symptoms predicted greater somatic symptoms as mediated by greater brooding in response to weekly negative events. Similarly, results indicated that greater baseline depressive symptoms significantly predicted greater brooding, greater brooding significantly predicted greater somatic symptoms at week eight, and the relationship between depressive and somatic symptoms significantly decreased when brooding was entered into the model. Specifically, the effect of baseline depressive symptoms on somatic symptoms at week eight decreased by 25.58% when brooding was in the model, suggesting that the effect of depressive symptoms on somatic symptoms is partially mediated by greater brooding in response to weekly negative events.

## 4. Discussion

Findings supported that depressive and somatic symptoms reciprocally predicted each other over the study period, and this relationship was partially mediated by greater dampening in response to weekly positive events and greater brooding in response to weekly negative events. Shared cognitive mechanisms between symptoms clusters have been theoretically proposed, but our analyses present the first statistical examination of the specific cognitive responses of dampening, positive rumination, and brooding in this reciprocal relationship. Contrary to our hypotheses, positive rumination did not mediate the relationship between depressive and somatic symptoms in either direction. This relationship was previously unexamined in somatic symptom literature but represented an established predictor of depressive symptoms in previous studies. Although rumination is a supported mechanism for the transmission of depressive and somatic symptoms [14, 20], positive rumination and brooding represented distinct effects in the current study. Because dampening and brooding appear to predict depressive and somatic symptom transmission by decreasing positive affect and increasing negative affect in response to events, positive rumination may exert a nonsignificant effect due to its protective increase in positive affect. Consequently, comorbid symptom transmission appears to be driven by limited positive affect and excessive negative affect that are translated into psychological and physical symptoms. Despite the nonsignificant impact of rumination in response to positive events, positive rumination is still indicated as a protective factor against depressive symptoms [35] and may represent an important mechanism in the prevention and treatment of isolated depressive disorders.

## 5. Conclusions

Depressive and somatic symptoms represent distinct but related sources of distress and impairment with global impacts on health. Consequently, identifying transdiagnostic cognitive mechanisms that transmit vulnerability is essential to disrupt maladaptive cycles and promote an integrated view of mind-body health. Findings implicate the reciprocal transmission of vulnerability between depressive and somatic symptoms through cognitive pathways, which is consistent with existing cognitive theories [12–14]. However, findings extend existing literature by proposing specific means of comorbid transmission and subsequent targets for cognitive intervention. For example, clinicians may prevent or interrupt the damaging effects of dampening and brooding by educating individuals on mindfulness techniques to consciously disengage from negative content. While positive rumination was not indicated as a significant cognitive mediator, alternative cognitive interventions that increase positive emotions such as mindfulness training [10] may be utilized as replacement cognitive activities to enhance protective tendencies.

As previously stated, depressive symptoms do not consistently precede the onset of somatic symptoms or vice versa [8]. Instead, dampening and brooding exert effects on both symptom categories by decreasing positive affect in response to positive events and increasing negative affect in response to negative events. Over time, dampening and brooding may create enduring patterns of high negative affect and low positive affect, which may be manifested as a combination of psychological and physical complaints. There are several potential explanations for how dampening and brooding transmit vulnerability between depressive and somatic symptoms. From a cognitive perspective, the negative cognitive focus provided by dampening and brooding may increase and reinforce confirmation biases that view the world pessimistically [36], which then increase the perceived severity of psychological and physical symptoms due to greater attentional focus on the negative. Depressive and somatic symptoms may become comorbid due to this bias because negative interpretations are generalized to psychological and physical sensations, and attentional focus seeks to confirm negative perceptions of comorbid symptoms. From a social perspective, cognitive intensification of negative emotions may prompt social withdrawal among individuals with depressive and somatic concerns [21, pp. 253–256], which further limits an individual's access to positive experiences and social support [37, 38]. Lack of social support consequently limits resources to manage distress, which may intensify and diffuse across psychological and physical domains. From a physiological perspective, dampening and brooding may activate and sustain sympathetic arousal throughout the body, which over time depletes cognitive, emotional, and physical resources and impedes recovery from existing psychological and physical conditions [39–41]. Whether the cognitive means of symptom transmission is due to confirmation bias, social withdrawal, and/or sustained sympathetic arousal, cognitive mechanisms are indicated as bridges between symptom clusters.

*Limitations and Future Directions.* Several study limitations warrant consideration. First, our sample of young adults captures a specific subpopulation that may not generalize to all adult populations. We selected this sample to examine somatic symptoms specifically among young adults because this age group has demonstrated greater psychological distress in combination with comorbid somatic symptoms compared to geriatric populations with similar comorbidities [28, 29]. However, future directions for research may include replication of the presented findings with more diverse samples. Second, our study represents a longitudinal diary design that provides weaker support for causality than an experimental paradigm. To address this weakness, we included multiwave measurements to establish temporal precedence for causality and theoretical rationales to justify our explanations of statistical relationships. Third, although our division of symptoms into depressive and somatic groupings reflects existing diagnostic categories, the high comorbidity between depressive and somatic symptoms imposes barriers that may constrain understanding of the continuous and overlapping nature of both categories. Despite our categorical investigation of depressive and somatic symptoms, we acknowledge that both clusters represent amalgamated patterns of distress and impairment. To address this concern, we selected the CES-D and MSPQ due to their distinct item content. Specifically, no CES-D items reference physical sensations and no MSPQ items reference emotions or cognition. While psychological and physical health symptoms necessarily coexist, our selected instruments provided clear distinctions between depressive and somatic symptoms. Finally, our study focused on the cooccurrence of somatic and depressive symptoms. Somatic symptoms also commonly cooccur with anxiety symptoms, and future research should examine mechanisms driving that cooccurrence as well.

As researchers and clinicians increasingly recognize the dynamic relationships between depressive and somatic symptoms, cognitive responses to positive and negative events offer mechanisms to reduce vulnerability and understand the transmission of comorbid symptom presentations. Future research is needed to investigate comorbid mechanisms of vulnerability transmission and acknowledge multiple diagnoses as the clinical norm. To this end, we present our findings to enhance appreciation of the reciprocal impact of psychological and physical health and emphasize the power of thinking in the transmission of comorbid vulnerabilities to depressive and somatic symptoms.

## Figures and Tables

**Table 1:** Variable correlations and descriptors.

Variable	1	2	3	4	5	6	M (SD)
(1) W1 depressive							14.47 (9.10)
(2) W1 somatic	0.54^*∗∗*^						18.29 (4.73)
(3) Avg. dampening	0.44^*∗∗*^	0.39^*∗∗*^					4.46 (1.39)
(4) Avg. positive rumination	−0.11^*∗*^	0.00	0.08				13.35 (3.56)
(5) Avg. brooding	0.46^*∗∗*^	0.39^*∗∗*^	0.63^*∗∗*^	0.17^*∗∗*^			9.52 (2.97)
(6) W8 depressive	0.47^*∗∗*^	0.43^*∗∗*^	0.50^*∗∗*^	0.00	0.51^*∗∗*^		12.92 (9.68)
(7) W8 somatic	0.41^*∗∗*^	0.57^*∗∗*^	0.42^*∗∗*^	0.03	0.43^*∗∗*^	0.59^*∗∗*^	16.60 (4.87)

*Note.* W: week; Avg.: average of weeks 2–7. ^*∗*^ *p* < 0.05; ^*∗∗*^ *p* < 0.01.

**Table 2:** Bootstrapping test of mediation effects predicting depressive symptoms.

	Standardized				
Path	*β*	SE	95% CI		*p*
			Lower	Upper	
*Combined model*					
*c*: somatic→depressive	0.08	0.02	0.05	0.11	0.003
*c*′: somatic→depressive	0.05	0.02	0.02	0.08	0.003

*Dampening*					
*α*: somatic→dampening	0.05	0.02	0.03	0.08	0.002
*β*: dampening→depressive	0.25	0.07	0.11	0.40	0.002
*c*′: somatic→depressive	0.06	0.02	0.03	0.09	0.003
*α* × *β*(*c* − *c*′)	0.01	0.00	0.05	0.22	0.001

*Positive rumination *					
*α*: somatic→positive rumination	0.02	0.01	0.00	0.04	0.109
*β*: positive rumination→depressive	−0.08	0.05	−0.17	0.01	0.106
*c*′: somatic→depressive	0.10	0.02	0.07	0.13	0.003
*α* × *β*(*c* − *c*′)	0.00	0.44	0.00	0.04	0.067

*Brooding*					
*α*: somatic→brooding	0.05	0.01	0.02	0.08	0.002
*β*: brooding→depressive	0.28	0.07	0.14	0.40	0.002
*c*′: somatic→depressive	0.06	0.02	0.03	0.09	0.004
*α* × *β*(*c* − *c*′)	0.01	0.06	0.06	0.23	0.001

**Table 3:** Bootstrapping test of mediation effects predicting somatic symptoms.

	Standardized				
Path	*β*	SE	95% CI		*p*
			Lower	Upper	
*Combined model*					
*c*: depressive→somatic	0.04	0.01	0.03	0.06	0.002
*c*′: depressive→somatic	0.03	0.01	0.01	0.05	0.002

*Dampening*					
*α*: depressive→dampening	0.04	0.01	0.02	0.06	0.003
*β*: dampening → somatic	0.16	0.07	0.02	0.31	0.026
*c*′: depressive→somatic	0.03	0.02	0.02	0.05	0.002
*α* × *β*(*c* − *c*′)	0.01	0.24	0.01	0.06	0.016

*Positive rumination *					
*α*: depressive→positive rumination	−0.02	0.01	−0.03	−0.01	0.005
*β*: positive rumination→somatic	0.00	0.05	−0.10	0.09	0.986
*c*′: depressive→somatic	0.05	0.01	0.03	0.07	0.002
*α* × *β*(*c* − *c*′)	0.00	0.10	−0.01	0.01	0.998

*Brooding*					
*α*: depressive→brooding	0.04	0.01	0.03	0.06	0.002
*β*: brooding→somatic	0.19	0.06	0.07	0.32	0.004
*c*′: depressive→somatic	0.03	0.01	0.02	0.05	0.002
*α* × *β*(*c* − *c*′)	0.01	0.06	0.01	0.07	0.002
